# Structure and Conformational Properties of d-Glucose/d-Galactose-Binding Protein in Crowded Milieu

**DOI:** 10.3390/molecules22020244

**Published:** 2017-02-06

**Authors:** Alexander V. Fonin, Sergey A. Silonov, Asiya K. Sitdikova, Irina M. Kuznetsova, Vladimir N. Uversky, Konstantin K. Turoverov

**Affiliations:** 1 Institute of Cytology of the Russian Academy of Sciences, Laboratory of Structural Dynamics, Stability and Folding of Proteins, Tikhoretsky av. 4, St. Petersburg 197046, Russia; alexfonin@incras.ru (A.V.F.); silonovsa25@yandex.ru (S.A.S.); asia28298@yandex.ru (A.K.S.); imk@incras.ru (I.M.K.); 2 Saint-Petersburg Technological Institute (Technical University), Moskovsky av. 26, Saint-Petersburg 190013, Russia; 3 Department of Biophysics, St. Petersburg State Polytechnical University, Polytechnicheskaya av. 29, St. Petersburg 195251, Russia; 4 Department of Molecular Medicine and Byrd Alzheimer’s Research Institute, Morsani College of Medicine, University of South Florida, Tampa, FL 33612, USA

**Keywords:** d-glucose/d-galactose-binding protein, macromolecular crowding, polymers, protein unfolding, protein folding, protein aggregation, intrinsic fluorescence, circular dichroism

## Abstract

Conformational changes of d-glucose/d-galactose-binding protein (GGBP) were studied under molecular crowding conditions modeled by concentrated solutions of polyethylene glycols (PEG-12000, PEG-4000, and PEG-600), Ficoll-70, and Dextran-70, addition of which induced noticeable structural changes in the GGBP molecule. All PEGs promoted compaction of GGBP and lead to the increase in ordering of its structure. Concentrated solutions of PEG-12000 and PEG-4000 caused GGBP aggregation. Although Ficoll-70 and Dextran-70 also promoted increase in the GGBP ordering, the structural outputs were different for different crowders. For example, in comparison with the GGBP in buffer, the intrinsic fluorescence spectrum of this protein was shifted to short-wave region in the presence of PEGs but was red-shifted in the presence of Ficoll-70 and Dextran-70. It was hypothesized that this difference could be due to the specific interaction of GGBP with the sugar-based polymers (Ficoll-70 and Dextran-70), indicating that protein can adopt different conformations in solutions containing molecular crowders of different chemical nature. It was also shown that all tested crowding agents were able to stabilize GGBP structure shifting the GGBP guanidine hydrochloride (GdnHCl)-induced unfolding curves to higher denaturant concentrations, but their stabilization capabilities did not depend on the hydrodynamic dimensions of the polymers molecules. Refolding of GGBP was complicated by protein aggregation in all tested solutions of crowding agents. The lowest yield of refolded protein was achieved in the highly concentrated solutions of PEG-12000. These data support the previous notion that the influence of macromolecular crowders on proteins is rather complex phenomenon that extends beyond the excluded volume effects.

## 1. Introduction

Protein folding has been intensively studied for the past 50 years. Currently, most of the data related to folding of different proteins were obtained as a result of in vitro experiments. As a rule, these experiments were performed in dilute solutions, but the processes of in vitro and in vivo folding are significantly different [1]. Firstly, protein folding in the cell is intimately associated with protein biosynthesis. When a nascent polypeptide chain leaves the ribosome, the protein begins to fold immediately, giving rise to the cotranslational folding process. Secondly, there is always a danger of formation of incorrect intramolecular contacts. Therefore chaperones and foldases (i.e., enzymes catalyzing *cis-trans* isomerization of proline residues and formation of ‘correct’ disulfide bonds) are required for correct protein folding in the cellular environment [1]. Thirdly, protein folding in vivo occurs in highly crowded environment; i.e., under conditions of minimal free space and permanent steric contacts of a protein undergoing folding process with other macromolecules.

Conditions mimicking macromolecular crowding in vitro are created using solutions containing high concentrations of inert polymers (so called crowding agents) such as polyethylene glycol (PEG), Dextran, Ficoll, etc. [2,3,4,5,6]. Concentrated solutions of these polymers are used for imitation of excluded volume effects; i.e., for generation of conditions under which volume occupied by molecules of crowding agents is not available for other macromolecules. As a consequence of mutual impenetrability of solute molecules, steric interactions between tested biological object and solvent molecules significantly increase in such conditions. Relationship between hydrodynamic dimensions of studied objects and crowding agents is one of the factors defining the “effectiveness” of a macromolecular crowder to affect different biochemical processes including protein folding. It is believed that the highest effectiveness of crowders on biochemical processes can be achieved under conditions where the target macromolecule and the crowding agent possess comparable hydrodynamic dimensions [7].

One of the main approaches for studying protein folding in vitro is the examination of protein structure and folding/unfolding processes by various biophysical methods [8]. This allows characterization of a conformational transition between native and unfolded states of a protein needed for the evaluation of the protein molecule stability. As a rule, the increase in protein stability is typically observed in the solutions of crowding agents [9,10,11]. One of reason for such stabilization is the increase in the difference of free energy between the native and unfolded states of protein due to the decrease in the conformational entropy of an unfolded polypeptide chain caused by the excluded volume effects [12,13]. However, several recent studies showed that this is not always the case, and some crowding agents are capable of destabilization of some proteins [14]. Decrease in the conformational entropy of a polypeptide chain and compaction of the protein structure in solutions of crowding agents could also promote increase in the number of non-native contacts between amino acid residues. This can cause protein aggregation in vitro especially during the presses of protein refolding when various partially folded intermediate can be formed [15,16].

The main goal of this work was to study the folding/unfolding reaction of d-glucose/d-galactose-binding protein (GGBP) from *E. coli* under conditions of macromolecular crowding. GGBP is a well-studied periplasmic ligand-binding monomeric protein that activates high affinity transport of glucose and galactose through MglABC transporter, promotes chemotaxis towards these sugars, and participates in the cooperative interactions between bacteria (quorum sensing) by binding with chemoreceptor Trg from *E. coli* [17]. The molecular mass of GGBP is 32 kDa, and its amino acid sequence has 309 residues. The spatial structure of this protein is organized in the N- and C-terminal domains with α/β/α fold linked by three strands commonly referred to as a hinge region that form the sugar-binding site. The active center of GGBP is formed by aromatic amino acids Trp 183 and Phe 16 [17]. Stacking interactions between these residues and sugar molecules make dominating contribution to the protein-ligand interaction [18]. It is known that the guanidine hydrochloride (GdnHCl)-induced unfolding of GGBP in diluted buffer solutions can be described as a two-state unfolding and is completely reversible [19]. On the other hand, the thermal denaturation GGBP is complicated by protein aggregation.

It is thought that macromolecular crowding exerts its greatest effects when the tested protein and crowders feature comparable hydrodynamic dimensions [7]. The hydrodynamic radius of GGBP is 28 Å [20]. For this reason the polymers differing in hydrodynamic dimensions and chemical nature were chosen as crowding agents: PEG-600 (a crowder with the average molecular mass of 600 Da, polymerization degree *n* = 14, and *R* = 5.6 Å), PEG-4000 (a crowding agent with an average molecular mass of 4000 Da, polymerization degree *n* = 91, and *R* = 16.5 Å), PEG-12000 (a crowder with an average molecular mass of 12,000 Da, polymerization degree *n* = 273, and *R* = 31 Å), the glucose-based polymer Dextran-70 (a crowding agent with an average molecular mass of 70,000 Da and *R* = 58 Å), and sucrose-based polymer Ficoll-70 (a crowder with an average molecular mass of 70,000 Da and *R* = 40 Å). 

## 2. Results and Discussion

### 2.1. The GGBP Structure under Crowding Conditions

**Figure 1:**
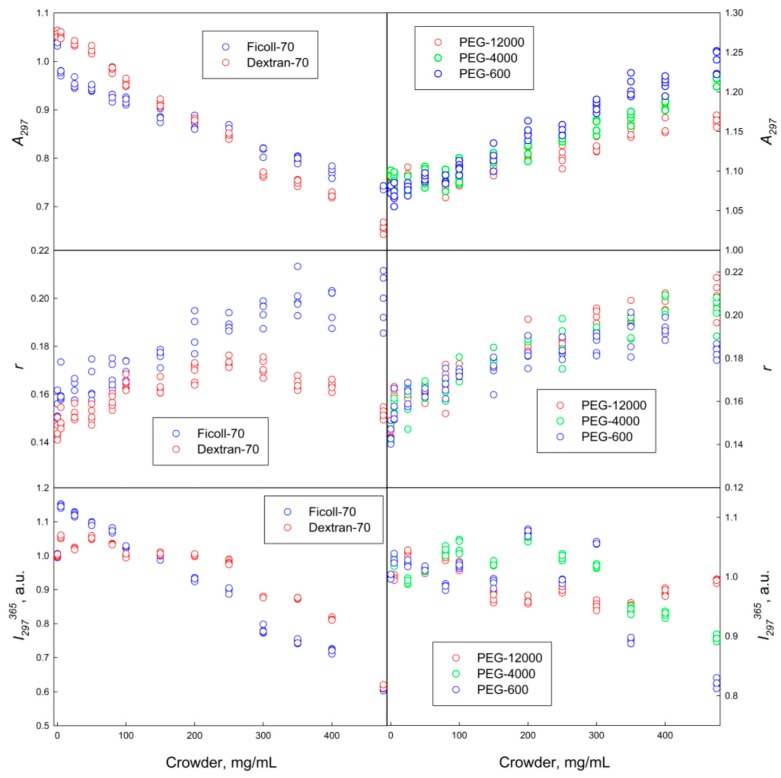
Characteristics of the GGBP intrinsic fluorescence in the presence of different crowding agents. The excitation wavelength was 297 nm.

Our analysis revealed that GGBP adopts different conformations in solutions of crowding agents of different chemical nature (polymers of ethylene glycol and sugars). Figure 1 shows that regardless of molecular mass, the increasing PEG concentrations induced the short-wave shift of protein fluorescence spectrum and the increase in its fluorescence of anisotropy. This indicates some PEG-induced compaction of GGBP, likely due to the excluded volume effects. 

**Figure 2:**
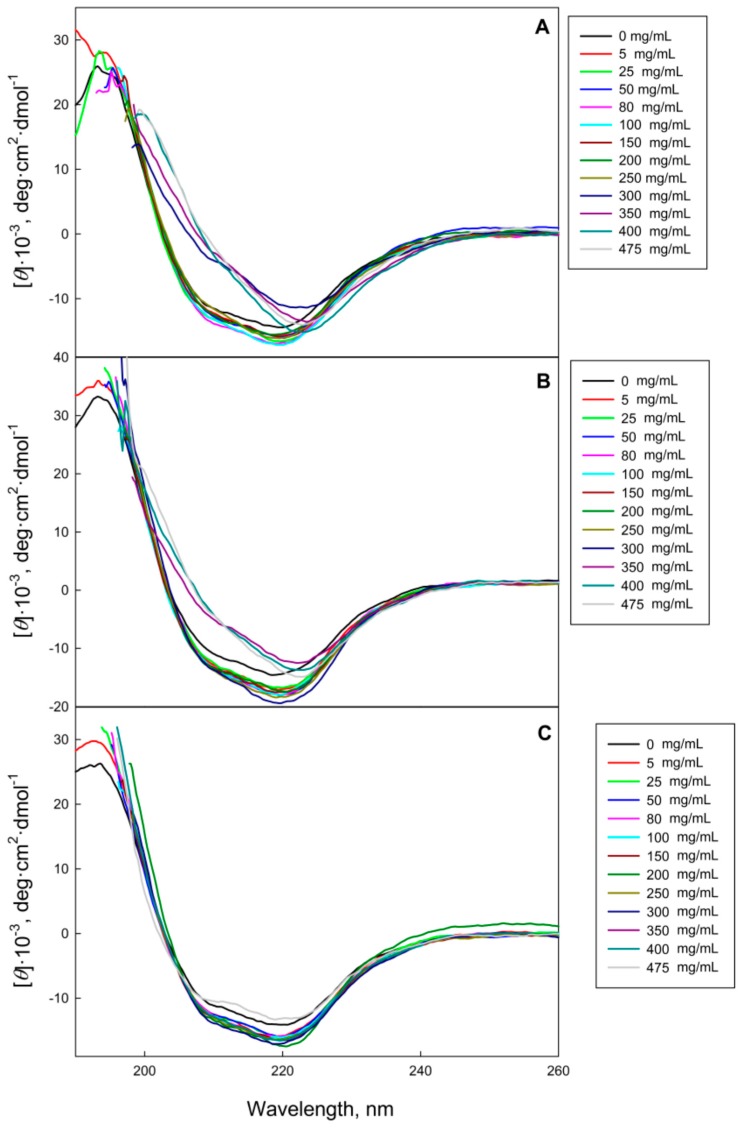
Far-UV CD spectra of GGBP in the presence of PEG of different molecular weight. (**A**): PEG-12000; (**B**): PEG-4000; (**C**): PEG-600.

Figure 2 shows that the molar ellipticity in the far-UV circular dichroism (CD) spectrum of GGBP increases with the increase in PEG concentration to 250 mg/mL in solutions of all tested PEGs. This indicates some increase in the amounts of ordered secondary structure under crowding conditions. Further increase in concentration of PEG-12000 and PEG-4000 promoted changes in the shape of far-UV CD spectra of GGBP. This may be due to the protein aggregation.

**Figure 3:**
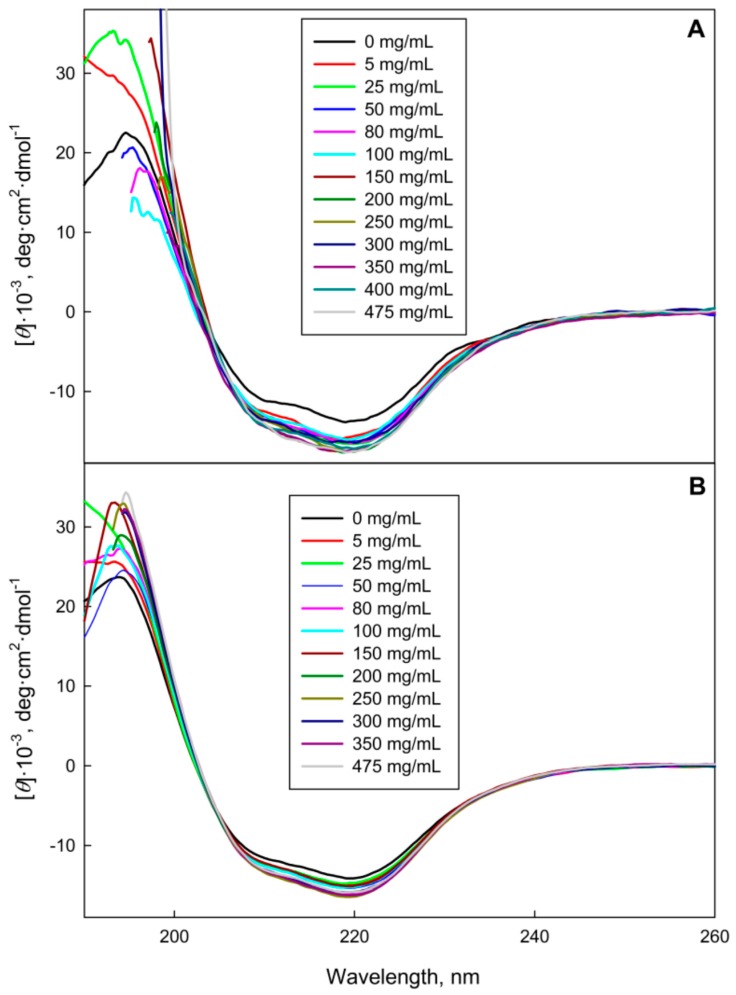
Far-UV CD spectra of GGBP in the presence of Ficoll-70 (**A**) and Dextran-70 (**B**).

GGBP characteristics in Dextran-70 and Ficoll-70 solutions were somewhat different from the spectral characteristics of this protein in PEG solutions. In fact, Figure 1 and Figure 3 show that the increase in the Dextran-70 and Ficoll-70 concentrations promoted long-wave shift of the GGBP intrinsic fluorescence spectrum and induced some increase in the amount of ordered secondary structure of this protein. Curiously, anisotropy of intrinsic fluorescence of GGBP monotonically increases with the increase in Ficoll-70 concentration, whereas the analogous dependence of intrinsic fluorescence anisotropy of GGBP on Dextran-70 concentration shows a maximum in the vicinity of 250 mg/mL of polymer. Since Dextran-70 and Ficoll-70 are polymers of glucose and sucrose, respectively, so one cannot exclude the possibility of binding of these polymers to the GGBP active center. This is in line with the previous work, where the possibility of specific interactions of maltose-binding protein (MBP, which is a structural analog of GGBP) with Ficoll-70 was reported [21]. It is known that the interaction of GGBP with glucose is accompanied by the increase in the content of ordered secondary structure of this protein, by the compaction of its tertiary structure, the decrease in the accessibility of GGBP tryptophan residues to solvent, and is also manifested by a small but reproducible short-wave shift of the intrinsic fluorescence spectrum of GGBP [19]. These observations on the structural causes of the glucose binding to GGBP seem to contradict to the data found in our study; i.e., the long-wave shift of the GGBP fluorescence spectrum in the Dextran-70 and Ficoll-70 solutions. To shed more light on this issue, we examined the accessibility of the GGBP tryptophan residues to solvent using the acrylamide-induced quenching of intrinsic fluorescence of GGBP in solutions of PEGs, Dextran-70 and Ficoll-70. Results of these analyses are reported in Table 1.

Table 1 shows that in solutions of all crowding agents analyzed in this study, the accessibility of GGBP tryptophan residues to solvent is noticeably decreased in comparison with solvent accessibility of protein tryptophan residues in buffer solution. The values of Stern-Volmer constant measured for GGBP in the presence of crowders are similar to that recorded for a protein in the presence of glucose. These data indicate that the compaction of protein tertiary structure takes place in solutions of PEG, Ficoll-70, Dextran-70, and glucose. Since the local microenvironment of GGBP tryptophan residues is sufficiently polar [22], the long-wave position of the GGBP fluorescence spectrum in solutions of Dextran-70 and Ficoll-70 could be explained by the crowder-induced decrease in distance between the quenching groups of amino acid residues of GGBP and tryptophan residues of this protein because of compaction of its tertiary structure.

### 2.2. Unfolding/Refolding of GGBP in Crowded Milieu

**Figure 4:**
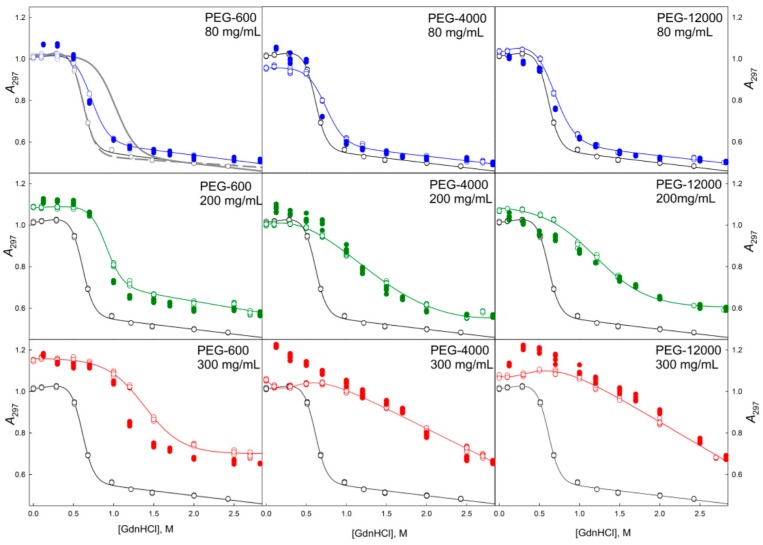
Unfolding/refolding transitions of GGBP induced by GdnHCl in the presence of different concentrations of PEGs of various molecular weights recorded as the GdnHCl dependencies of parameter *A* = *I*_320_/*I*_365_. The excitation wavelength was 297 nm. The unfolding of GGBP is represented by open symbols, whereas refolding is shown by closed symbols. The black curve is equilibrium GdnHCl-induced unfolding of GGBP in the absence of crowding agents. The gray curve in the upper left panel represents the equilibrium GdnHCl-induced unfolding transition of the glucose bound GGBP. The gray dashed curve in the same plot shows the equilibrium GdnHCl-induced unfolding of the GGBP sample prepared by the pre-incubation in 2.5 M GdnHCl solution followed by the extensive dialysis.

It is known that in the absence of crowding agents, the GdnHCl-induced unfolding of GGBP is described as a two-state reversible process. Complex formation of GGBP with glucose resulted in the significant shift of the unfolding transition to larger GdnHCl concentrations (Figure 4, upper left panel). On the other hand, unfolding curve of the GGBP pre-incubated in 2.5 M GdnHCl solution and regenerated after extensive dialysis GGBP coincided with the unfolding curve of the GGBP apo-form (Figure 4, upper left panel). These observations indicate that the protein purification procedure used in our study generates the glucose-free form of GGBP.

**Figure 5:**
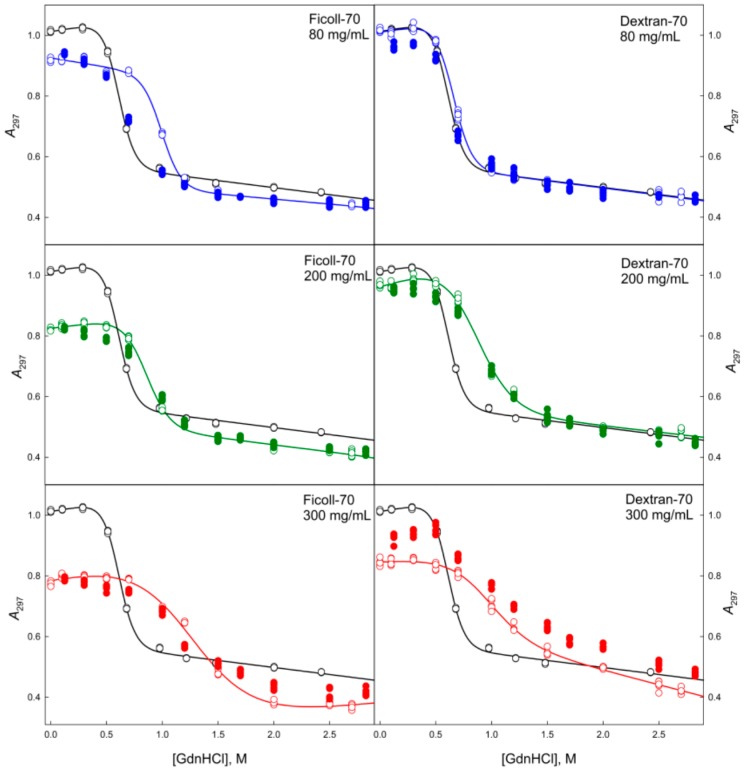
Unfolding/refolding transitions of GGBP in the presence of different concentrations of Ficoll-70 and Dextran-70 recorded by the dependence of parameter *A* = *I*_320_/*I*_365_ on GdnHCl concentration. The excitation wavelength was 297 nm. The designations as in Figure 4.

**Figure 6:**
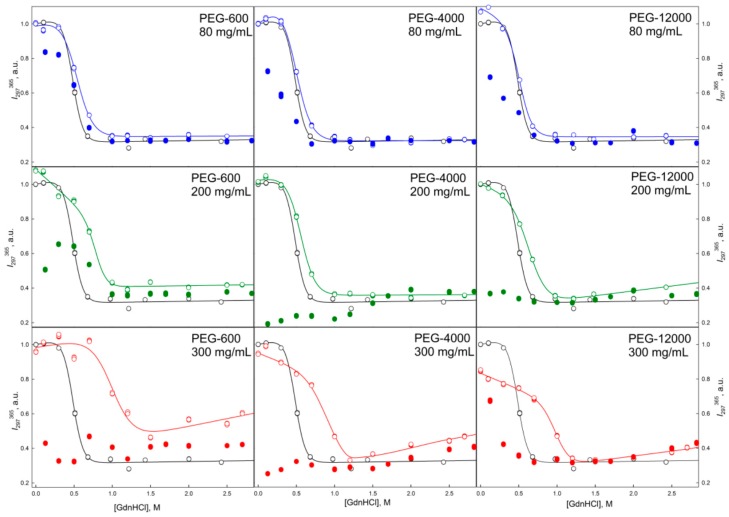
Unfolding/refolding transitions of GGBP in the presence of different concentrations of PEGs with different molecular weight recorded as the dependence of the intrinsic fluorescence intensity of GGBP on GdnHCl concentration. The excitation wavelength was 297 nm, the emission wavelength was 365 nm. Other designations as in Figure 4.

**Figure 7:**
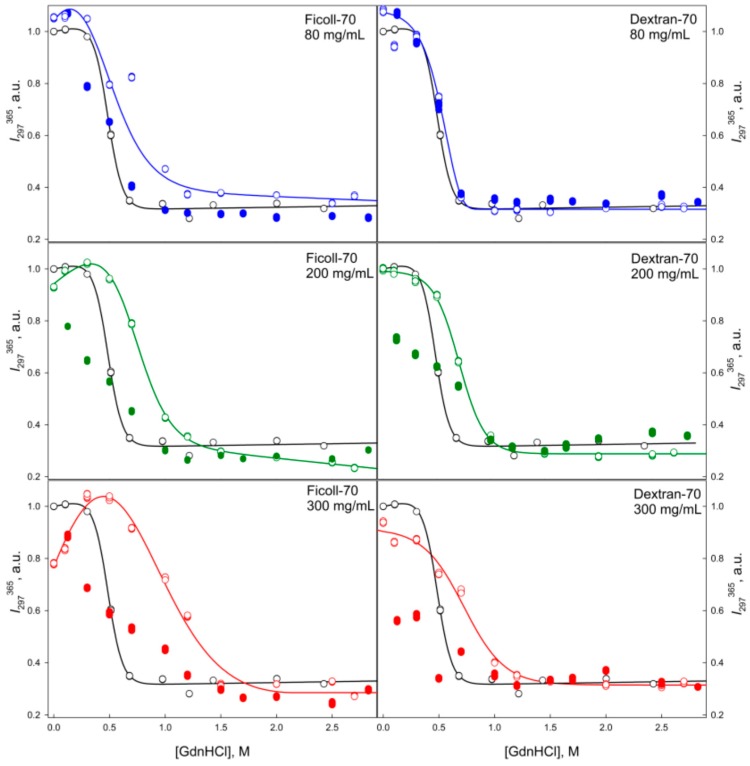
Unfolding/refolding transitions of GGBP in the presence of different concentrations of Ficoll-70 and Dextran-70 recorded as the dependence of the intrinsic fluorescence intensity of GGBP on GdnHCl concentration. The excitation wavelength was 297 nm, emission wavelength was 365 nm. Other designations as in Figure 4.

**Figure 8:**
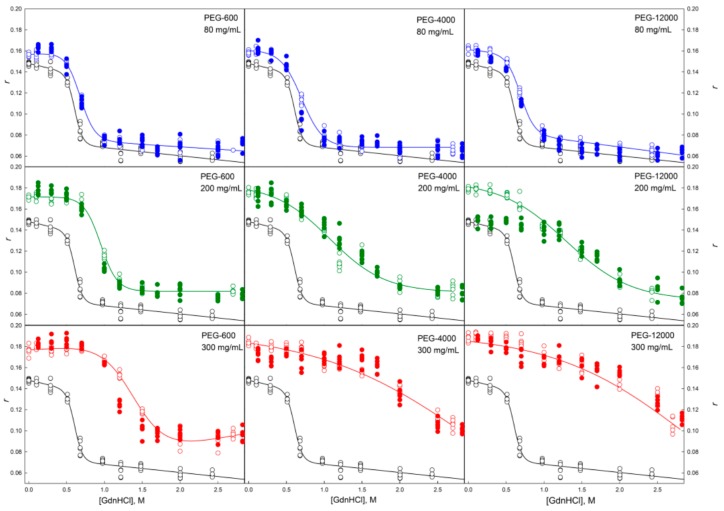
Unfolding/refolding transitions of GGBP in the presence of different concentrations of PEG of different molecular weight recorded by the dependence of the anisotropy of intrinsic fluorescence of GGBP on GdnHCl concentration. The excitation and emission wavelengths were 297 and 365 nm, respectively. Other designations as in Figure 4.

**Figure 9:**
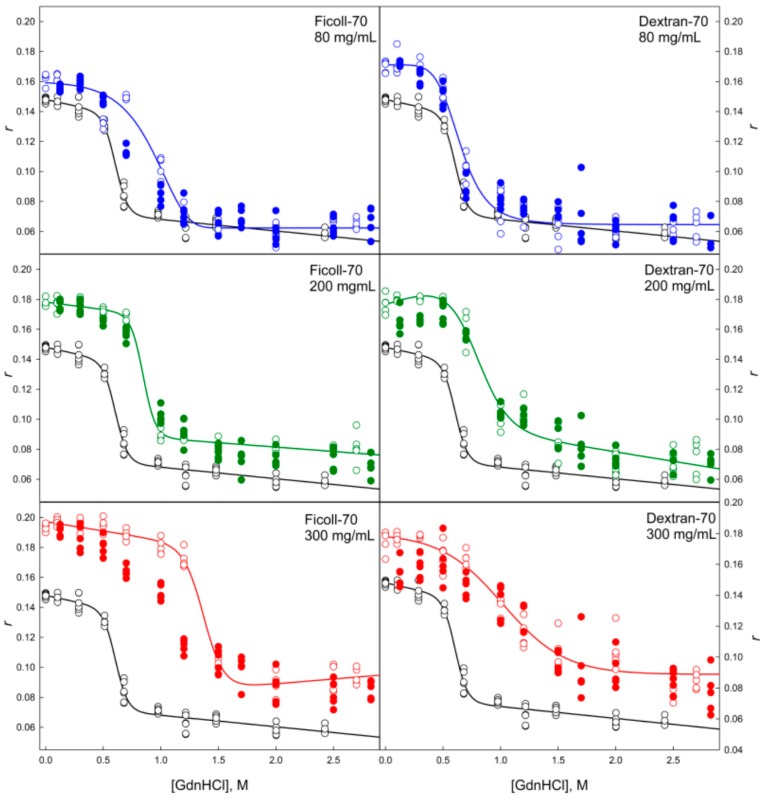
Unfolding/refolding transitions of GGBP in the presence of different concentrations of Ficoll-70 and Dextran-70 recorded as the dependence of the anisotropy of intrinsic fluorescence on GdnHCl concentration. The excitation wavelength was 297 nm, the emission wavelength was 365 nm. Other designations as in Figure 4.

**Figure 10:**
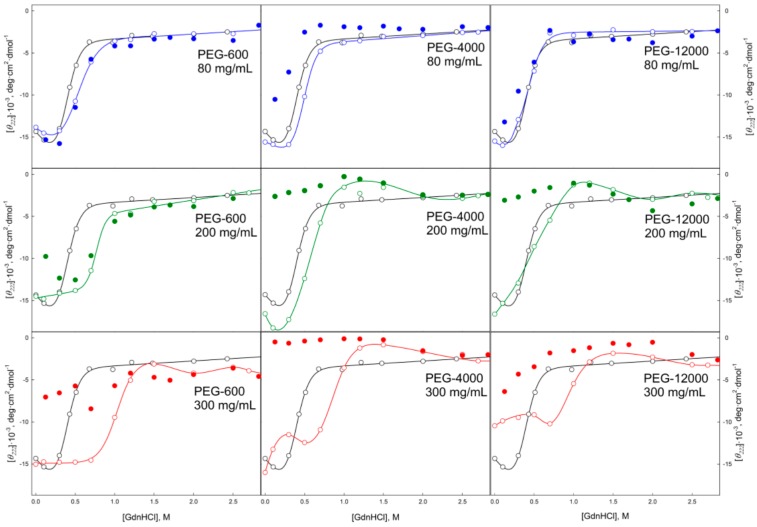
Unfolding/refolding transitions of GGBP in the presence of different concentrations of PEGs with different molecular weight recorded as dependence of GGBP molar ellipticity at 222 nm on GdnHCl concentration. The designations as in Figure 4.

**Figure 11:**
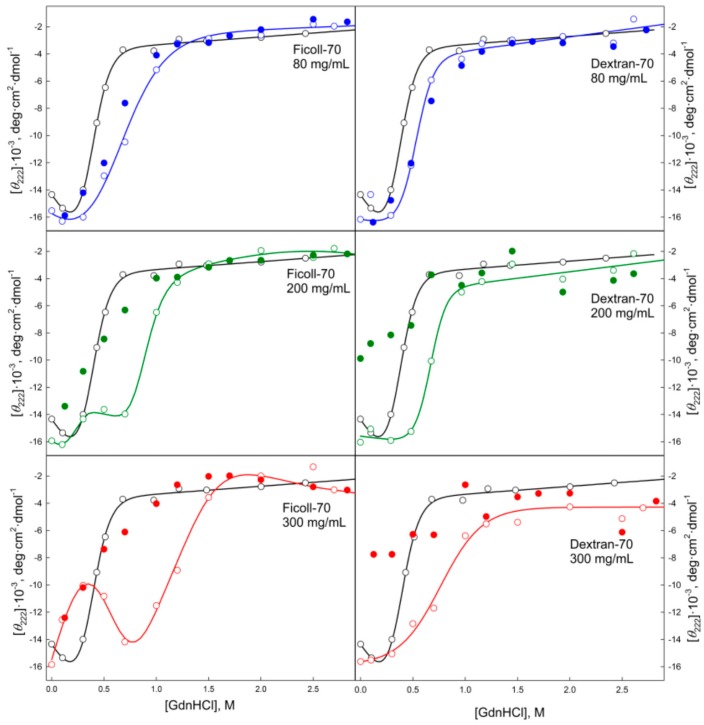
Unfolding/refolding transitions of GGBP in the presence of different concentrations of Ficoll-70 and Dextran-70 recorded by the dependence of the molar ellipticity at 222 nm on GdnHCl concentration. Other designations as in Figure 4.

Data presented in Figure 4, Figure 5, Figure 6, Figure 7, Figure 8, Figure 9, Figure 10 and Figure 11 clearly show that in solutions containing 80 mg/mL of Dextran-70, Ficoll-70, and PEG (regardless of its polymerization degree), the unfolding curves of GGBP are shifted toward higher GdnHCl concentrations in comparison with the positions of the corresponding curves in the absence of crowding agents.

In this work, we conducted two series of experiments to gain the information on the peculiarities of the GGBP unfolding/refolding in crowded milieu. In the first series, we investigated the concentration effect of the selected crowding agent on the process of protein unfolding. In the second series, experiments were conducted at fixed crowder concentrations to analyze the effects of crowding agents of different molecular mass on the unfolding/refolding reaction. As expected, the increase in the excluded volume caused by the increase in the concentrations of the crowding agent (PEG-600) resulted in a shift of the GdnHCl-induced unfolding curve of GGBP toward higher denaturant concentrations. On the other hand, although increasing the size of the crowding agent among PEG-600, PEG-4000 and PEG-12000 at constant weight concentration of a given crowder (80 mg/mL) also increases the excluded volume (volume occupied by PEGs are 6%, 23% and 50%, respectively), no shifts of the unfolding curves were caused by the increase of the excluded volume in this case. Based on these observations it was concluded that the differences in the unfolding-refolding behavior of a target protein in concentrated polymer solutions and in dilute buffered solutions due not only to the excluded volume effect, but may be caused by some other factors as well.

Earlier, several in vitro and in silico studies reported the results of investigation of the effect of macromolecular crowding on proteins with the topology similar to the structural organization of GGBP [23,24,25,26,27,28]. For example, it has been shown that increasing Ficoll concentration increased the stability and folding rate of the *B. burgdorferi* VlsE, as well as caused a change in the shape of this protein from an ellipsoidal to spherical form, leading to the exposure of a hidden antigenic region [23]. It was also established that the conditions of molecular crowding can promote significant shift of the equilibrium between the calmodulin conformers toward more compact forms [26]. It was also shown that under the conditions of molecular crowding phosphoglycerate kinase (PGK) became more compact, and there was a significant increase in the functional activity and stability of PGK under these conditions [25]. In general, our data are correlated with the results reported in these studies, since it is shown that highly concentrated solutions of crowding agents can promote compaction of the target proteins, increase the orderliness of the protein structure, and increase the stability of the investigated proteins toward thermal and chemical denaturation and unfolding. However, the fact that the GGBP conformational stability was only minimally dependent on the ratio between the hydrodynamic dimensions of the protein and crowding agents suggested the existence of the excluded volume-independent mechanisms of the effect of highly concentrated polymer solutions on the GGBP stability. It is likely that these mechanisms are related to the changed structure of water in the concentrated solutions of crowding [29,30]. The GdnHCl dependences of the GGBP intensive fluorescence characteristics (such as parameter *A* and fluorescence anisotropy) measured at protein refolding coincide with unfolding processes, indicating good reversibility of the unfolding transition (see Figure 4, Figure 5, Figure 8 and Figure 9).

When comparing the unfolding and refolding dependencies of the molar ellipticity and fluorescence intensity of GGBP on GdnHCl concentration, hysteresis is observed in solutions of PEG-4000 and PEG-12000 (see Figure 6 and Figure 10), indicating the quasi-stationary nature of the obtained dependences and slow establishment of equilibrium in the solutions of PEG-4000 and PEG-12000. Further increase of concentration of crowding agents regardless of their hydrodynamic dimensions and chemical structure promoted further shift of the GGBP unfolding curves to larger GdnHCl concentrations (Figure 4, Figure 5, Figure 6, Figure 7, Figure 8, Figure 9, Figure 10 and Figure 11). Also, a significant decrease in the cooperativity of unfolding transition was observed in solutions of PEG-4000 and PEG-12000, likely indicating the non-equilibrium nature of the obtained dependences.

In order to confirm this hypothesis, the kinetics of the GGBP unfolding in solutions of different concentrations of PEGs, Dextran-70 and Ficoll-70 was studied for 4 weeks (data not shown). This analysis revealed that at all tested time intervals, the CD signal of GGBP decreased and the protein fluorescence spectrum was red-shifted in PEG solutions. However, these effects were not observed in solutions containing Dextran-70 and Ficoll-70. It is likely that these observations reflect the presence of gradual aggregation/destabilization of protein in PEG solutions. The GGBP unfolding curves measured in the presence of high concentrations of PEG and Ficoll-70 as dependencies of the molar ellipticity and fluorescence intensity on GdnHCl concentration have local maximum and minimum in the vicinity of 1.2–1.7 M GdnHCl (Figure 10 and Figure 11), respectively. 

Such effects were not observed in the Dextran-70 solutions. Decrease in the fluorescence intensity and CD signal in this region of denaturant concentrations could indicate the decrease in the effective protein concentration due to aggregation of GGBP under these conditions. Furthermore, when protein concentration was high, precipitation was observed under these conditions. These findings are consistent with the current views on a higher degree of compaction of an unfolded protein in crowded milieu compared to that in dilute buffers [31,32,33,34,35,36].

GGBP refolding curves measured in the presence of highly concentrated solutions of crowding agents (200 and 300 mg/mL) in a form of GdnHCl dependences of parameter *A* and protein fluorescence anisotropy coincide with the unfolding curves recorded as GdnHCl-induced changes of these GGBP characteristics (see Figure 4, Figure 5, Figure 8 and Figure 9). However, unfolding and refolding curves recorded as GdnHCl dependencies of the GGBP molar ellipticity and intensity of protein intrinsic fluorescence were significant different in the presence of high concentrations of all tested polymers (see Figure 6, Figure 7, Figure 10 and Figure 11). 

Even in solutions with low denaturant concentrations, values of the CD signal and fluorescence intensity of GGBP obtained after protein refolding under these conditions were close to the values of the corresponding characteristics of unfolded GGBP. Apparently, during GGBP refolding, the increase in the concentration of crowding agents promoted formation of protein aggregates that lead to the decrease in the effective GGBP concentration, which was reflected in low CD and fluorescence signals. The lowest yield of refolded protein was observed at high concentrations of PEG-4000 and PEG-12000.

## 3. Materials and Methods

### 3.1. Materials

PEG-600, PEG-4000, PEG-12000, Ficoll-70, Dextran-70, guanidine hydrochloride (Sigma, St. Louis, MO, USA) were used without further purification. To determine the GdnHCl concentration, we relied on the measurement of the refraction coefficient using Abbe refractometer (LOMO, St. Petersburg, Russia).

*E. coli* strain K-12 (*F^+^mgl503 lacZ lacY*^+^*recA1*) carrying an *mglB* gene deletion transformed with a pTz18u-*mglB* vector was primarily used for the obtaining of GGBP wild type. Upon induction with d-fructose, the expression efficiency of the GGBP protein was rather low.

The recombinant protein yield in this system does not exceed 5–8 mg/L of culture. Therefore, for the increase in the expression levels, the nucleotide sequence of *mglB* gene was optimized and the gene was recloned into a pET-11d plasmid with the T7 promoter (Stratagene, La Jolla, CA, USA) using *NcoI* and *BglII* restriction sites. Specific forward and reverse primers were used to insert new restriction sites and a polyhistidine tag at the C-terminal region of the target protein. Site-directed mutagenesis was performed with the Quik-Change mutagenesis kit (Stratagene) using primers encoding sequences corresponding to the desired amino acid substitutions. Plasmids were isolated from bacterial cells using plasmid DNA isolation kits (Omnix, St. Petersburg, Russia). Primer purification was performed using either reverse-phase chromatography or electrophoresis in a polyacrylamide gel [37].

pET-11d plasmid encoding GGBP was used to transform *E. coli* BL21(DE3) cells. The expression of the protein was then induced by adding 0.5 mM isopropyl-beta-d-1-thiogalactopyranoside (IPTG; Nacalai Tesque, Kyoto, Japan). Bacterial cells were cultured for 24 h at 37 °С. Recombinant proteins were purified using Ni^2+^-agarose packed in the His-GraviTrap columns (GE Healthcare, Chicago, IL, USA). Protein purification was controlled using denaturing SDS-electrophoresis in 15% polyacrylamide gel.

The experiments were performed in solutions with protein concentration of 0.2 mg/mL. All solutions were based on the sodium phosphate buffer at pH 7.4. All experiments were conducted at 23 °C.

However, at the beginning of our work with recombinant GGBP, we carried out systematic analysis aimed at the investigation of the structure of this protein in different conformational states. Among these preliminary studies, we compared the structural characteristics of the purified protein and apo-protein produced by the standard procedure for elimination of the tightly bound ligands by partial unfolding of the protein at low GdnHCl concentrations (1.5–2.5 M) followed by the extensive dialysis. We found that the structural characteristics of GGBP were identical in both cases. Furthermore, in one of our papers [19] we have shown that the complexation of GGBP with glucose causes a shift in the middle of the transition between the native and unfolded states of protein from 0.4 M GdnHCl to 0.9 M GdnHCl.

### 3.2. Methods

#### 3.2.1. Steady-State Fluorescence Spectroscopy 

The fluorescence experiments were carried out using Cary Eclipse (Agilent, Santa Clara, CA, USA) spectrofluorimeter. The measurements were made at 23 °C with 10 mm × 10 mm cells (Starna, Atascadero, CA, USA). The fluorescence intensity of tryptophan residues was corrected for the primary inner filter effect [38]:

(1) $$F_{0}\left(\lambda_{ex}\right)=F\left(\lambda_{ex}\right)/W$$

where *W* is the factor that corrects measured total fluorescence intensity for so-called primary inner filter effect.

Because the fluorescence measurements were performed using the Cary Eclipse spectrofluorimeter with horizontal slits, the value of correction factor *W* was calculated using the following ratio:

(2) $$W=\frac{\left(1-10^{-A_{\Sigma }}\right)}{A_{\Sigma }}$$

where *A*_Σ_ is the total absorbance of exciting light in the solution. In work [38] it was shown that the corrected in such manner value of the total fluorescence intensity is a product of the absorbance *A_FL_* to the quantum yield of fluorescence *q* when one fluorescent substance is present in solution.

The excitation wavelength for the intrinsic protein fluorescence was 297 nm. The position and form of the UV fluorescence spectra were characterized by the parameter *A* = *I*_320_/*I*_365_, where *I*_320_ and *I*_365_ are the fluorescence intensities measured at emission wavelengths of 320 and 365 nm, respectively. The values of parameters *A* and the fluorescence spectra were corrected using the instrument’s spectral sensitivity.

#### 3.2.2. Dynamic Quenching of Intrinsic Fluorescence

The study of dynamic quenching of the intrinsic UV fluorescence of wild type GGBP by small quencher acrylamide in sodium phosphate buffer, 0.1 M GdnHCl and 0.4 M urea was carried out for evaluating the accessibility of protein tryptophan residues to solvent under these conditions. The protein intrinsic fluorescence was excited at 297 nm and total intrinsic UV fluorescence (from 300 to 450 nm) was registered. 

The quenching constants were obtained using Stern-Volmer equation written for total fluorescence intensity:

(3) $$\frac{F\left(\left[0\right]\right)}{F\left(\left[Q\right]\right)}=\frac{W([0])}{W([Q])}\left(1+K_{SV}[Q]\right)$$

where *F*([0]) is the total fluorescence in the absence of quencher, *F*([*Q*]) is the total fluorescence in the presence of quencher at a concentration [Q], *W*([0]) is correction factor *W* in the absence of quencher, *W*([*Q*]) is correction factor *W* in the presence of quencher, $K_{SV}$ is Stern–Volmer constant; i.e., the rate constant of fluorescence quenching due to collisions of quencher molecules with fluorophore molecules in the excited state. Here, it is assumed that $A_{FL}([0])$ = $A_{FL}([Q])$. It is necessary to bear in mind that the acrylamide absorbs at the wavelength of excitation 297 nm. Therefore, the recorded fluorescence intensity should be amended, relating to changes in *W* with the increase in the quencher concentration.

#### 3.2.3. Circular Dichroism Measurements 

CD spectra were obtained using a Jasco-810 spectropolarimeter (Jasco, Tokyo, Japan). Far-UV CD spectra were recorded in a 1 mm path length cell from 260 nm to 190 nm with a step size of 0.1 nm. Near-UV CD spectra were recorded in a 10 mm path length cell from 320 nm to 250 nm with a step size of 0.1 nm. For all spectra, an average of three scans was obtained. CD spectra of the appropriate buffer solution were recorded and subtracted from the protein spectra.

## 4. Conclusions

The results obtained in this study suggest that some compaction and increase in the ordering of GGBP structure are observed under molecular crowding conditions in vitro regardless of the hydrodynamic dimensions and chemical nature of the model crowding agents. Differences in the positions of the GGBP intrinsic fluorescence spectra in PEG, Ficoll-70, and Dextran-70 solutions indicate that these conditions create different microenvironments for the GGBP tryptophan residues, and, likely, induce different protein conformations. Compaction of the GGBP spatial stricture was observed in all tested polymer solutions. We also show that the increase in the concentrations of all polymers in tested solutions induces a noticeable shift in the GGBP unfolding transition toward higher GdnHCl concentrations, suggesting a crowding-induced increase in protein stability. This stabilization can be a result of the decrease in the total available volume in the considered systems. The shift of unfolding curves did not significant depend on the hydrodynamic dimensions of crowding agents. Crowded environment promoted aggregation of GGBP in the presence of 1.2–1.7 M GdnHCl and high concentrations of PEG-4000, PEG-12000, and Ficoll-70. Refolding of GGBP in solutions of all crowding agents used in this study was complicated by protein aggregation. These observations confirm the necessity of chaperons and other cellular machinery for correct protein folding in vivo.

## Figures and Tables

**Table 1:** Some spectroscopic characteristics of GGBP in crowding milieu.

Crowding Agent	*K_SV_*, M^−1^	*A*_297_	*r*	[*θ*]_222_·10^−3^, deg·cm^2^·dmol^−1^
PEG-12000	2.1	1.12	0.20	−11
PEG-4000	2.2	1.16	0.18	−18
PEG-600	1.7	1.18	0.18	−16
Ficoll-70	1.3	0.82	0.19	−16
Dextran-70	1.7	0.77	0.17	−15
No crowding agents *	4.7	1	0.15	−14
Glucose *	1.9	1	0.16	−15

All experiments were performed in buffer solutions (see Materials and Methods). Concentration of all crowding agents was 300 mg/mL. * For comparison, the same experiments were conducted in the absence of crowding agents and in solutions of glucose in concentration of 300 mg/mL.
